# Supplementary material for: Whole-Genome Sequencing-Based Characteristics in Extended-Spectrum Beta-Lactamase-Producing Escherichia coli Isolated from Retail Meats in Korea
Source: Microorganisms. 2020 Apr 2;8(4):508. doi: 10.3390/microorganisms8040508 (PMC7232390; doi:10.3390/microorganisms8040508)
Supplement: Supplementary file 1 [file microorganisms-08-00508-s001.zip › Figure S1.docx]

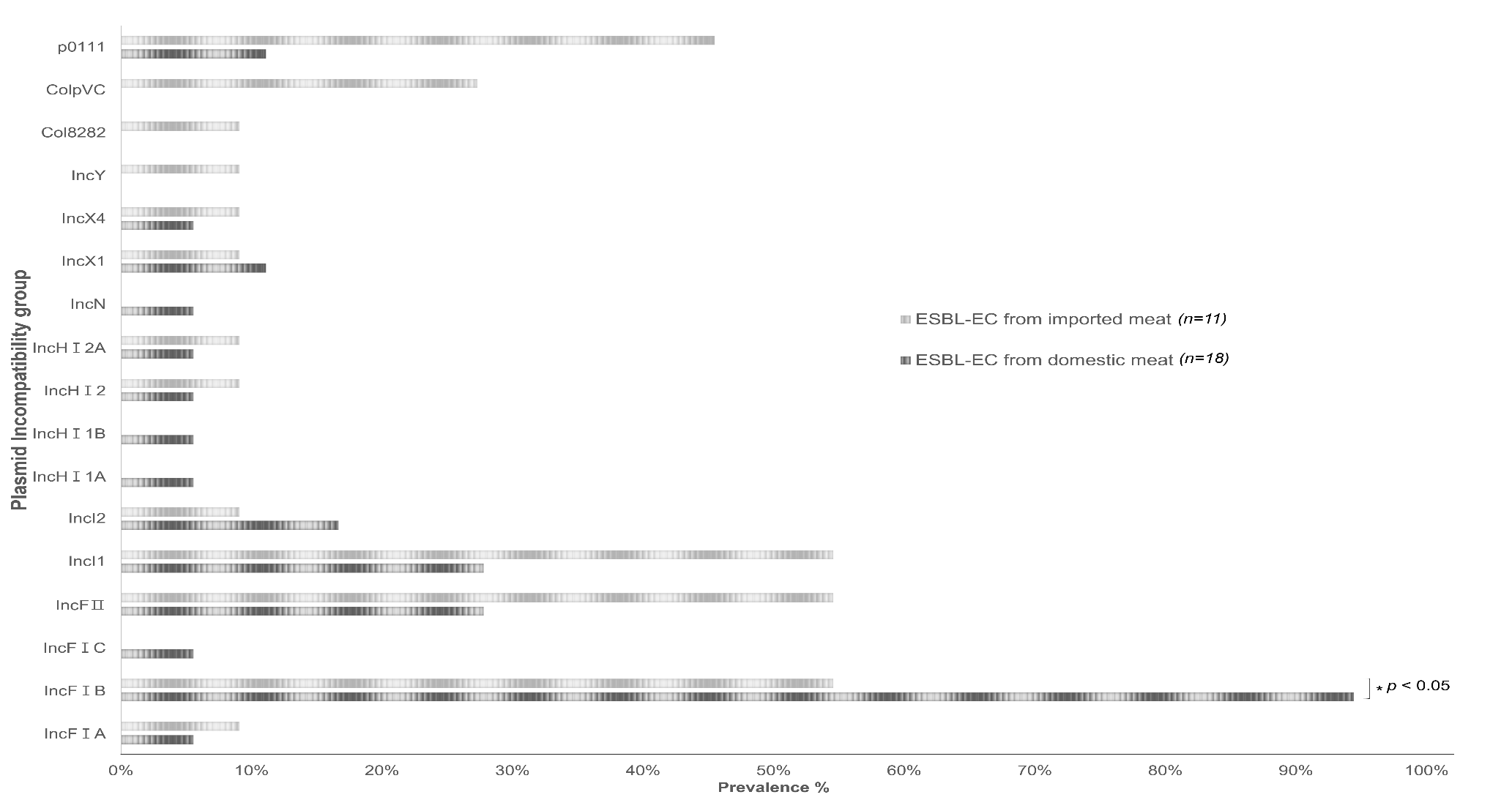


**Figure S1.** Distribution of plasmid replicons in 29 ESBL-EC from retail raw meats in Korea. * indicates significant difference (*P* < 0.05) between two groups in each prevalence of plasmid replicon.
