# Supplementary material for: Whole-Genome Sequencing-Based Characteristics in Extended-Spectrum Beta-Lactamase-Producing Escherichia coli Isolated from Retail Meats in Korea
Source: Microorganisms. 2020 Apr 2;8(4):508. doi: 10.3390/microorganisms8040508 (PMC7232390; doi:10.3390/microorganisms8040508)
Supplement: Supplementary file 1 [file microorganisms-08-00508-s001.zip › Figure S2.docx]

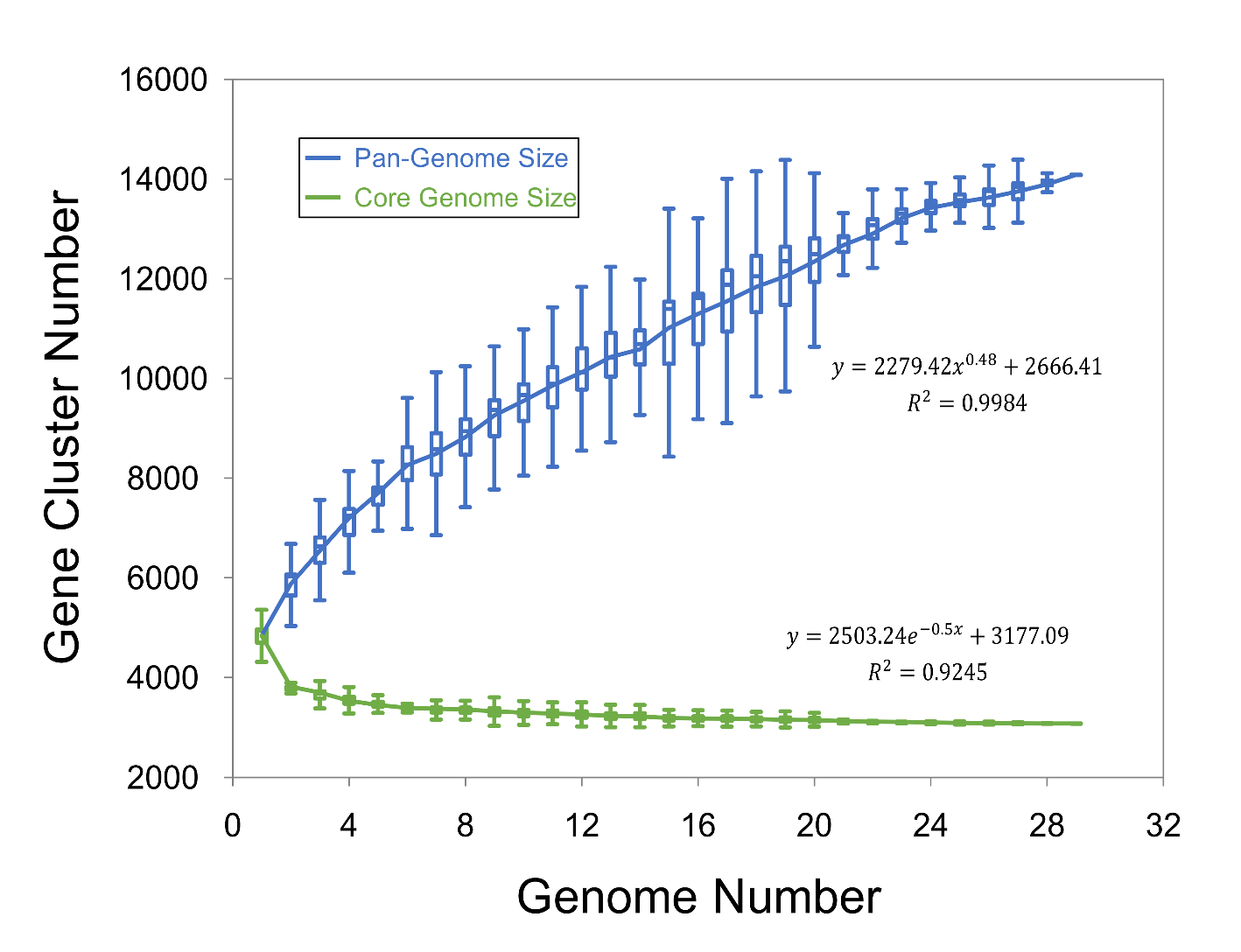


**Figure S2.** Pan-genome profile of 29 ESBL-EC isolates. Gene accumulation curves of the pan-genome (blue) and core-genome (green) were plotted as a function of the number of genomes sequentially added (*n=29*), using PanGP. For the pan-genome, the accumulation curve (blue) shows the fit ($r^{2}=0.9984$) of the power-law regression model. The pan-genome size is calculated at 14,094 genes, and its trajectory (blue) shows characteristics of an open pan-genome.
