# Supplementary material for: Whole-Genome Sequencing-Based Characteristics in Extended-Spectrum Beta-Lactamase-Producing Escherichia coli Isolated from Retail Meats in Korea
Source: Microorganisms. 2020 Apr 2;8(4):508. doi: 10.3390/microorganisms8040508 (PMC7232390; doi:10.3390/microorganisms8040508)
Supplement: Supplementary file 1 [file microorganisms-08-00508-s001.zip › Table S1(corrected).docx]

Table 1. List of antimicrobial agents, their subclasses, MIC range tested concentration and breakpoints for susceptibility testing.

| Antimicrobial  subclass | Antimicrobial Agents  (Abbreviation) | MIC range tested concentration  (µg/mL) | Breakpoints  (µg/mL) | References |
| --- | --- | --- | --- | --- |
| Aminoglycosides | Gentamicin (GEN) | 1-64 | ≥16 | CLSI |
|  | Streptomycin (STR) | 16-128 | ≥32 | NARMS |
| Aminopenicillin | Ampicillin (AMP) | 2-64 | ≥32 | CLSI |
| Beta-lactam/beta-lactamase inhibitor combinations | Amoxicillin/ clavulanic acid (AmC) | 2/1-32/16 | ≥32/16 | CLSI |
| Cephamycin | Cefoxitin (FOX) | 1-32 | ≥32 | CLSI |
| Cephalosprin III | Ceftiofur (CTF) | 0.5-8 | ≥8 | NARMS |
|  | Ceftazidime (CAZ) | 1-16 | ≥16 | CLSI |
| Cephalosprin IV | Cefepime (FEP) | 0.25-16 | ≥16 | CLSI |
| Carbapenem | Meropenem (MEM) | 0.25-4 | ≥4 | CLSI |
| Fluoroquinolone | Ciprofloxacin (CIP) | 0.12-16 | ≥4 | CLSI |
| Folate pathway inhibitors | Trimethoprim/ Sulfamethoxazole (SXT) | 0.12/2.38-4/76 | ≥4/76 | CLSI |
| Phenicols | Chloramphenicol (CHL) | 2-64 | ≥32 | CLSI |
| Polymyxins | Colistin (COL) | 2-32 | >2 | EUCAST |
| Tetracyclines | Tetracycline (TET) | 2-128 | ≥16 | CLSI |
| Quinolone | Nalidixic acid (NAL) | 2-128 | ≥32 | CLSI |

1. Clinical Laboratory Standards Institute [1]
2. The National Antimicrobial Resistance Monitoring System [2]
3. The European Committee on Antimicrobial Susceptibility testing [3]
